# Verification and diagnostic evaluation of the RealStar^®^ Middle East respiratory syndrome coronavirus (N gene) reverse transcription-PCR kit 1.0

**DOI:** 10.2217/fmb-2019-0067

**Published:** 2019-07-02

**Authors:** Leonie-Sophie Hecht, Angeles Jurado-Jimenez, Markus Hess, Hussein El Halas, Gregor Bochenek, Hala Mohammed, Fahd Alzahrani, Mohammed O Asiri, Rami Hasan, Aref Alamri, Sultan Alotaibi

**Affiliations:** 1 aItona Diagnostics GmbH, Hamburg, Germany; 2 Ministry of Health, Riyadh Regional Laboratory, Riyadh, Saudi Arabia; 3 King Fahad Medical City, Riyadh, Saudi Arabia

**Keywords:** diagnostics, emerging diseases, MERS-CoV, molecular diagnostics, preparedness, real-time RT-PCR, WHO diagnostic scheme

## Abstract

**Aim:**

We report the diagnostic evaluation of a confirmatory reverse transcription-PCR (RT-PCR) kit targeting the Middle East respiratory syndrome coronavirus (MERS-CoV) N gene.

**Material & methods:**

33 patient samples from two collections sites in Riyadh, Saudi Arabia, which were pre-characterized via real-time RT-PCR targeting MERS-CoV *orf1a* and *upE*, and were tested using the MERS-CoV N gene, as a confirmatory assay. This diagnostic procedure follows a two-step diagnostics scheme, recommended by the WHO.

**Results:**

18/33 samples tested positive, 11/33 tested negative for MERS-CoV RNA and 2/33 showed uncertain results.

**Conclusion:**

The results suggest, that the RealStar^®^ MERS-CoV (N gene) RT-PCR kit 1.0 can be considered a suitable and reliable confirmatory assay in combination with the RealStar MERS-CoV RT-PCR kit 1.0 according to the diagnostic scheme recommended by WHO.

Emerging infectious diseases are becoming an increasingly important issue not only in tropic and subtropic regions but also in the northern hemisphere. Due to changes in their geographical distribution viruses like West Nile virus, Zika virus or others are gaining grounds [1]. Not too long ago, in 2003, Canada was overwhelmed by an emerging disease outbreak with approximately 400 incidences and 44 deaths, caused by the SARS virus. This virus was brought into the country by an infected woman traveling from Hong Kong to Toronto [2,3].

Nearly a decade later, in April 2012 a SARS-like disease was described on the Arabian Peninsula, caused by an, until then, unknown *Betacoronavirus*. Later that year this ‘novel coronavirus’ was officially named the Middle East respiratory syndrome coronavirus (MERS-CoV) [4].

MERS-CoV is an enveloped positive-sense single-stranded RNA virus. Its genome (∼30 kb [5]), encodes four structural proteins [6].

MERS is a zoonotic disease that might originate from (African) bats as host species, but because MERS-CoV never has been isolated directly from bats [7], the virus most likely evolved and spread from bats to camels, which became a new reservoir for MERS-CoV. Most human cases have been linked (directly or indirectly) to contact with camels or to human-to-human transmission [8].

The clinical spectrum of MERS-CoV infection ranges from no symptoms or mild respiratory symptoms to severe acute respiratory disease and death. Pneumonia and gastrointestinal symptoms, including diarrhea, may also occur [9]. In elderly people, and those with chronic diseases, such as renal disease, cancer, chronic lung disease or diabetes, the virus seems to cause more severe disease [6,9]. Although MERS is a disease mainly found in adults, there are also known cases involving children, with severe illness and fatalities caused by MERS-CoV [10].

The awareness and preparedness for such emerging disease outbreaks increased after the SARS-CoV outbreaks. Preparedness includes access to up-to-date case reports of ongoing outbreaks, based on active and passive surveillance programs [1], and being ready with sufficient diagnostic protocols and tools [11]. This had taken effect in case of the first MERS-CoV outbreak on the Arabian Peninsula. The WHO and other healthcare authorities had proven their ability to respond quickly by providing the first laboratory diagnostics protocol based on real-time reverse transcription-PCR (RT-PCR) in September 2012 [12,13].

In 2015, MERS-CoV emerged in South Korea. It had been brought into the country by a traveler, coming back from the Middle East. One traveler initiated an outbreak with 186 cases and 38 fatalities [14]. As the man did not have any contact with either camels or camel products or any persons with respiratory syndromes, MERS-CoV was not considered as the cause of his symptoms at the beginning. Not until many other diagnostic tests came up negative and the pneumonia progressed, the patient was tested for MERS-CoV and came up positive. Further epidemiological examinations revealed that the human-to-human transmission in the Korean outbreak required either only 10 min exposure in an emergency room where MERS-CoV infected persons were hospitalized or a 2-min talk with an infected person [14].

This outbreak situation showed impressively that a single (initially) missed case has the potential to cause a huge and nationwide outbreak. Reliable diagnostic tools are essential in order to be able to detect potential threats in time and to initiate proper patient management, isolate infected patients and to protect uninfected persons [14].

The WHO recommends a protocol for the molecular diagnostic detection of MERS-CoV. This protocol gets constantly revised. In the current version of the protocol WHO recommends a two-step diagnostic scheme: first, screening samples with an assay targeting the upstream region of the E gene, the so-called *upE* assay, followed by a confirmatory assay targeting a sequence within the open reading frame 1a or 1b, the so-called *orff1a* or *orf1b* assay. For samples with discrepant results for *upE* and *orf1a/orf1b*, WHO recommends sequencing of a third target, either the RNA dependent RNA polymerase *RdRp* or the *N gene*. A modified version of this two-step diagnostic scheme is shown in Figure 1. Routine diagnostic laboratories might not be able to perform a sufficient sequencing analysis and therefore rather perform a third RT-PCR targeting either *rdrp* or *N gene*.

**Figure 1. ;:**
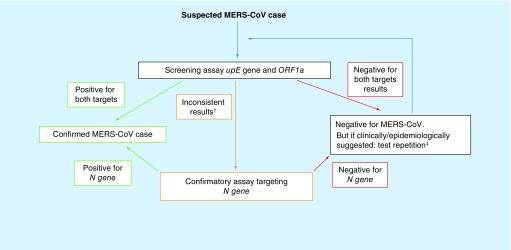
Schematic depictionof the two-step diagnostic scheme for the detection of MERS-CoV specific RNA by screening for two different sequence targets, e.g. *upE* and *orf1a*. In case of inconsistent results of the screening assays, conformational sequencing or RT-PCR of a third target should be performed (modified from the diagnostic scheme recommended by WHO in 2012 [19]). ^†^Positive only for one of the two targets. ^‡^Where applicable: use of different/additional specimen type.

Validated, European Conformity-*In Vitro* Diagnostics (CE-IVD) marked and Emergency Use Authorization (EUA) labeled (emergency use authorized by US FDA) kits for the RT-PCR based detection of MERS-CoV specific RNA, that allow the diagnosis of MERS-CoV according to the WHO protocol (Figure 1), are commercially available.

Altona Diagnostics GmbH (Hamburg, Germany), well known for their broad portfolio of RT-PCR-based kits for the detection of emerging and tropical viruses and pathogens, provides two kits for the detection of MERS-CoV. The RealStar^®^ MERS-CoV RT-PCR kit 1.0 consists of two independent assays, one targeting the region upstream of the E gene (*upE*) and the second targeting the open reading frame 1a (*orf1a*). In addition, altona Diagnostics GmbH has recently developed and validated the RealStar MERS-CoV (N gene) RT-PCR kit 1.0 targeting the N gene. This assay is intended to be used to reanalyze samples with discordant results in the *upE* and *orf1a* assays. Assay characteristics and analytical performance data can be found in the Instructions for use (IFU) of the assays (http://altona-diagnostics.com/en/support/downloads.html).

Here, we present data on the diagnostic validation of the RealStar MERS-CoV (N gene) RT-PCR kit 1.0. The diagnostic testing was obtained by analyzing 33 samples collected from suspected MERS-CoV infected patients that had been tested with the RealStar MERS-CoV RT-PCR kit 1.0. These samples had previously either been characterized as MERS-CoV negative or positive for both targets, *upE* and *orf1a*, or positive in only one target.

## Material & methods

### Clinical samples

Thirty-three samples (either nasal swabs or nasopharyngeal aspirates) were collected from suspected MERS-CoV infected patients at two different sites in Riyadh, at the King Fahad Medical City Pathology and Clinical Laboratory Medicine Administration and the Regional Laboratory (Riyadh, Saudi Arabia) in 2015.

### Nucleic acid extraction

The nucleic acids of the samples were extracted using the MagNA Pure LC 2.0, MagNA Pure LC Total Nucleic Acid Isolation kit, external lysis protocol and MagNa Pure 96 Total NA Isolation kit and the Pathogen Universal 200 3.1 protocol (Roche Life Science, IN, USA). The sample volume was 200 μl, the elution volume 50 μl according to the manufacturer manual for urine/cerebrospinal fluid/swabs. The sample volume was 200 μl, the elution volume 50 μl.

### Real-time PCR

The samples were analyzed using the CE-IVD marked RealStar MERS-CoV RT-PCR kit 1.0 (altona Diagnostics GmbH) according to the manufacturer’s ‘instruction for use’ (http://altona-diagnostics.com/en/support/downloads.html) on the LightCycler^®^ 480 Instrument II (Roche) according to the manufacturers manual and default settings.

The RealStar MERS-CoV RT-PCR kit 1.0 is comprised of two independent detection assays, one targeting a region upstream of the E gene (*upE*) and the other targeting the open reading frame 1a (*orf1a*). Each assay contains an internal control (IC), which can be used to monitor the efficiency of the nucleic acid extraction process and possible inhibitory effects during RT-PCR.

All samples, from both testing sites, were stored at -20°C and reanalyzed retrospectively in 2018 at Molecular Microbiology Department, Pathology and Clinical Laboratory Medicine Administration for the purpose of validating the RealStar MERS-CoV (N gene) RT-PCR kit 1.0 (altona Diagnostics GmbH) as a confirmatory assay according to the diagnostic scheme proposed by the WHO (Figure 1). The RealStar MERS-CoV (N gene) RT-PCR kit 1.0 is comprised of the N gene specific detection system. Assay characteristics and analytical performance characteristics can be found in the IFU of the kit (http://altona-diagnostics.com/en/support/downloads.html). The analytical sensitivity of the RealStar MERS-CoV (N gene) RT-PCR kit 1.0, determined by Probit analysis is 0.71 copies/μl (CI: 0.43–1.62 copies/μl).

An IC, which can be used as a nucleic acid extraction control and/or as an RT-PCR inhibition control, is part of the kit. The IC is a heterologous RT-PCR system, with an *in vitro* transcript (IVT) of artificial sequence as target molecule. The IC was used and analyzed according to the IFU of the kit (http://altona-diagnostics.com/en/support/downloads.html).

### Cross-reactivity testing of the RealStar MERS-CoV (N gene) RT-PCR kit 1.0

Absence of cross-reactivity was tested by analyzing genomic DNA/RNA of different organisms either related to MERS-CoV, showing the same prevalence or causing similar symptoms (Table 1) using the RealStar MERS-CoV (N gene) RT-PCR kit 1.0 on the CFX96™ Deep Well RT-PCR Detection System (Bio-Rad, CA, USA) instrument. The DNA/RNA of each pathogen was analyzed in three replicates.

**Table 1. ;:** Pathogens tested for cross-reactivity with the RealStar^®^ Middle East respiratory syndrome coronavirus (N Gene) reverse transcription-PCR kit 1.0.

Pathogen	Material	Catalog number (provider)	Concentration (provider)	Concentration tested
Adenovirus	Genomic DNA	VR-1 (ATCC)	TCID50 ≥10^3.0^ per 0.2 ml	Dilution 1:1000
*Bordetella pertussis*	Genomic DNA	DSM 5571 (DSMZ)	79.9 ng/μl	Dilution 1:200
*Chlamydophila pneumoniae*	Genomic DNA	DSM 19748 (DSMZ)	112.5 ng/μl	Dilution 1:1000
*Haemophilus influenzae*	Genomic DNA	DSM 4690 (DSMZ)	253.5 ng/μl	Dilution 1:1000
hMPV	Genomic RNA	0810164CFHI (Zeptometrix, hMPV 27 Type A2)	TCID50 ≥10^6.10^ U/ml	Eluate 1:10 diluted
Human coronavirus NL63	Genomic RNA	University Bonn	Not available	Dilution 1:10
Human coronavirus 229E	Genomic RNA	University Bonn	Not available	Dilution 1:10
Human coronavirus OC43	Genomic RNA	University Bonn	Not available	Dilution 1:10
Human coronavirus HKU1	Genomic RNA	University Bonn	Not available	Dilution 1:10
Influenza A virus	Genomic RNA	0810252CFHI (Zeptometrix)	Not available	Dilution 1:10
Influenza B virus	Genomic RNA	0810255CFHI (Zeptometrix)	Not available	Dilution 1:10
*Klebsiella pneumoniae*	Genomic DNA	33495 (ATCC)	Not available	Eluate 1:10 diluted
*Legionella pneumophila*	Genomic DNA	DSM 7513 (DSMZ)	163 ng/μl (diluted 1:500)	Dilution 1:500
*Mycobacterium tuberculosis*	Genomic DNA	25618D-2	≥2 μg/46 μl	Dilution 1:100
*Mycoplasma pneumoniae*	Genomic DNA	DSM 22911 (DSMZ)	Not available (diluted 1:10)	Dilution 1:100
Parainfluenza virus	Genomic RNA	VR-94 (ATCC)	TCID50 ≥10^3.0^ per 0.2 ml	Dilution 1:100
Respiratory syncytial virus	Genomic RNA	VR-26D (ATCC)	600 ng per 100 μl	Dilution 1:10
Rhinovirus	Genomic RNA	NCPV (0112169)	Not available	Dilution 1:1000
SARS-CoV	Genomic RNA	University Bonn	Not available	Dilution 1:10
*Streptococcus pneumoniae*	Genomic DNA	DSM 20566 (DSMZ)	260 ng/μl (diluted 1:1000)	Dilution 1:1000
*Streptococcus pyogenes*	Genomic DNA	DSM20565 (DSMZ)	Not available	Dilution 1:10

ATCC: American-type culture collection; CoV: Coronavirus; DSMZ: Deutsche Sammlung von Mikroorganismen und Zellkulturen.

## Results

In 2015, a total number of 33 suspected MERS-CoV specimens had been collected at two different laboratory sites in Riyadh, Saudi Arabia. These samples were analyzed using the RealStar MERS-CoV RT-PCR kit 1.0.

11 samples had been tested at the Molecular Microbiology Department, Pathology and Clinical Laboratory Medicine Administration (Table 2; sample numbers 1–11). Ten out of these eleven samples showed positive signals with the *of1a* assay. Eight samples had shown according results using the *upE* assay but two samples were positive with only one assay but negative with the other. By definition of the WHO these samples are equivocal or uncertain. One suspected MERS-CoV specimen was tested negative for MERS-CoV RNA with both assays (Table 2).

**Table 2. ;:** Diagnostic evaluation of suspected Middle East respiratory syndrome coronavirus specimens.

Sample number	RealStar MERS-CoV RT-PCR kit 1.0			RealStar MERS-CoV (N Gene) RT-PCR kit 1.0	
	*upE* specific (FAM)	*orf1a* specific (FAM)	Internal controls (VIC)	*N gene* specific (FAM)	Internal control (VIC)
2	33.39	34.52	Valid	34.05	Valid
3	32.75	37.47	Valid	36.20	Valid
4	30.00	29.84	Valid	30.85	Valid
5	34.92	38.77	Valid	38.92	Valid
6	34.90	35.89	Valid	37.22	Valid
7	31.00	31.94	Valid	31.26	Valid
8	17.31	17.46	Valid	20.36	Valid
**9**	**ND**	**28.72**	**Valid**	**32.67**	**Valid**
**10**	**ND**	**38.09**	**Valid**	**38.08**	**Valid**
11^†^	ND	ND	Valid	ND	Valid
12	ND	ND	Valid	ND	Valid
13	ND	ND	Valid	ND	Valid
14	ND	ND	Valid	ND	Valid
15	ND	ND	Valid	ND	Valid
16	ND	ND	Valid	ND	Valid
17	ND	ND	Valid	ND	Valid
18	ND	ND	Valid	ND	Valid
19	ND	ND	Valid	ND	Valid
20	ND	ND	Valid	ND	Valid
21	ND	ND	Valid	ND	Valid
22	32.00	32.00	Valid	34.97	Valid
23	30.00	30.00	Valid	33.00	Valid
24	28.00	29.00	Valid	30.59	Valid
25	26.00	26.00	Valid	31.93	Valid
26	28.00	29.00	Valid	31.03	Valid
27	34.00	35.00	Valid	37.39	Valid
28	25.00	24.00	Valid	30.45	Valid
29	15.00	15.00	Valid	19.86	Valid
30	28.00	29.00	Valid	30.62	Valid
**31**	**ND**	**37.00**	**Valid**	**ND**	**Valid**
32	25.00	29.00	Valid	29.47	Valid
**33**	**36.00**	**ND**	**Valid**	**ND**	**Valid**

Bold values highlight samples with inconsistent outcome.

† H1N1 positive.

FAM: MERS-CoV specific detection channel; MERS-CoV: Middle East respiratory syndrome coronavirus; ND: Not detected; RT-PCR: Reverse transcription-PCR; VIC: IC detection channel.

22 samples were analyzed at the regional laboratory (Table 2; sample numbers 12–33). Out of these 22 samples, 10 were tested positive for both targets, 2 were tested positive for only one target (equivocal by definition of the WHO) and 10 were tested negative for MERS-CoV RNA (Table 2).

In order to validate the RealStar MERS-CoV (N gene) RT-PCR kit 1.0 all sample eluates were retested retrospectively at Molecular Microbiology Department, Pathology and Clinical Laboratory Medicine Administration King Fahad Medical City in 2018. Therefore, the 22 specimens from the Regional Laboratory were transferred to the King Fahad Medical City Pathology and Clinical Laboratory Medicine Administration.

All 18 samples tested positive with both assays of the RealStar MERS-CoV RT-PCR kit 1.0 also tested positive with the RealStar MERS-CoV (N gene) RT-PCR kit 1.0.

All 11 samples tested negative with the RealStar MERS-CoV RT-PCR kit 1.0 also tested negative with the RealStar MERS-CoV (N gene) RT-PCR kit 1.0.

The two samples tested positive with only one of the assays of the RealStar MERS-CoV RT-PCR kit 1.0 at Molecular Microbiology Department, Pathology and Clinical Laboratory Medicine Administration, tested positive with the RealStar MERS-CoV (N gene) RT-PCR kit 1.0, too. But, the two samples tested positive with only one of the assays of the RealStar MERS-CoV RT-PCR kit 1.0 at the Regional Laboratory, tested negative with the RealStar MERS-CoV (N gene) RT-PCR kit 1.0.

None of the DNA/RNA of different pathogens (Table 1), either related to MERS-CoV, showing the same prevalence or causing similar symptoms as MERS-CoV, was tested positive for MERS-CoV N gene showing the excellent specificity of the RealStar MERS-CoV (N gene) RT-PCR kit 1.0.

## Discussion

Since its discovery in 2012 the MERS-CoV has caused 2279 confirmed cases in 27 countries with a total number of 806 fatalities worldwide [15]. From the very first (known) cases international expert teams worked together in order to investigate this emerging disease in more detail and to develop reliable diagnostic tools and protocols that can be used to contain the spread of the virus. Sequencing data were generated and released that enabled the development of specific molecular diagnostic assays to detect MERS-CoV and to distinguish it from other related human coronaviruses, like the SARS virus [2]. Up until today, there is no vaccine or specific treatment for MERS available; thus, early case recognition and isolation of infected persons is the only strategy to curb the spread of the virus. Since the virus is detectable shortly after the onset of symptoms and as other diagnostics, such as cell culture are slow and practicable only in specialized laboratories, the specific detection of MERS-CoV RNA via RT-PCR is the method of choice and recommended by WHO [6,16,17].

As illustrated in Figure 1, WHO recommends to test MERS-CoV suspected samples for two different genomic sequences, for example, a region upstream of the E gene (*upE*) and the open reading frame 1a (*orf1a*). According to this recommendations, a suspected MERS-CoV sample is considered positive when it is confirmed with two independent RT-PCR tests targeting different genomic regions [18]. A case with a positive PCR result for a single specific target without further testing but history of potential exposure is considered a probable case. The suggested course of action would be to either perform another PCR or sequencing of a third genomic region (Figure 1) [17,18], ideally with freshly extracted RNA from properly stored specimen [16]. False-negative results may occur due to poor quality of the specimen, collection of specimen late or very early in disease, inappropriate handling or shipment of specimen or due to technical reasons inherent to the performed test, such as virus mutation or PCR inhibition [19]. Proper specimen collection, transport conditions, extraction method and storages of eluates have a major impact on the quality of the RNA and therefore on reliable molecular diagnostic [16,20].

The CE-IVD marked RealStar MERS-CoV RT-PCR kit 1.0 [21], which is comprised of two independent assays, one targeting *upE* and the other targeting *orf1a*, was used at two different diagnostic laboratories in Riyadh, Saudi Arabia, to test 33 MERS-CoV suspected samples. Out of these 33 samples, 29 could be diagnosed as positive (n = 18) or negative (n = 11) for MERS-CoV RNA. But four samples (2 tested at Molecular Microbiology Department, Pathology and Clinical Laboratory Medicine Administration King Fahad Medical City and 2 tested at the regional laboratory) came up with equivocal results, positive with only one of the two assays (Table 2). These results demonstrate that equivocal results may occur occasionally.

There can be different reasons for equivocal results. As in this study all samples with equivocal results had valid IC signals, RT-PCR inhibition as an explanation for the negative results with one of the two assays can be excluded.

Differences in the sensitivity of the two assays might be another reason for equivocal results. But the analytical sensitivity of the two assays is similar [16,19]. Out of the four equivocal samples, three were positive with the *orf1a* assay and negative with the *upE* assay and one sample was vice versa, which also speaks against significant differences in sensitivity between the two assays. In most cases equivocal results occur when samples have virus concentrations close to the limit of detection of the assays leading to positive and negative results in a statistical manner. This is very likely the cause of the equivocal results for samples 10, 31 and 33.

Sample number 9 showed valid IC signals in both assays and a threshold cycle (Ct) value around 28 for *orf1a* but a negative result for *upE*.

The relatively low Ct values, of the positive *orf1a* result, suggest that the sample has a virus concentration well above the limit of detection of both assays. Most likely the equivocal result is therefore not an issue of sensitivity but caused by mutations within the *upE* target region which impairs *upE* assay performance.

With emerging viruses, when there is only limited sequence information available, a dual target strategy reduces the risk of false-negative results due to differences in the target sequences of the assays between different genotypes. This is especially important for RNA viruses, such as coronaviruses, which tend to have high mutation rates.

Following WHO’s recommendation for the diagnosis of MERS-CoV a confirmatory assay should be used for retesting equivocal samples.

In this study, we used the newly developed RealStar MERS-CoV (N gene) RT-PCR kit 1.0 to re-analyze all 33 samples (positive, negative and equivocal) at Molecular Microbiology Department King Fahad Medical City. For this purpose, the eluates of the 22 samples, initially analyzed at the Regional Laboratory, had been transferred to the Molecular Microbiology Department.

All samples tested positive with both assays of the RealStar MERS-CoV RT-PCR kit 1.0 were also tested positive with the RealStar MERS-CoV (N Gene) RT-PCR kit 1.0, and all samples originally tested negative could be confirmed negative with the RealStar MERS-CoV (N gene) RT-PCR kit 1.0.

Sample number 11, tested negative with all three MERS-CoV detection assays, was later confirmed to be positive for Influenza-A H1N1 RNA.

The two samples (9 and 10) with equivocal results tested at Molecular Microbiology Department, were positive with the N gene assay. Therefore, these samples can be considered positive for MERS-CoV specific RNA.

A different picture was presented with the samples transferred to Molecular Microbiology Department, Pathology and Clinical Laboratory Medicine Administration. The two equivocal samples showed negative results with the RealStar MERS-CoV (N gene) RT-PCR kit 1.0. Even though the IC signals were valid and therefore no inhibition of the RT-PCR was observed, no positive signal was detected in the specific channel. Therefore, according to the diagnostic protocol recommended by WHO these samples are considered negative for MERS-CoV. Since these samples had been stored for about 3 years and had been transported to Molecular Microbiology department, most recently, RNA decay might be a possible explanation of the absence of detectable MERS-CoV RNA in the two samples.

Both samples, one positive with the *orf1a* (Table 2; sample number 31) and one positive for *upE* (Table 2; sample number 33), had shown similar Ct values (>35) and therefore had most likely MERS-CoV RNA concentrations at the limit of detection of the two assays. Assays targeting the N gene are known to be slightly less sensitive in comparison to assays targeting *upE* and *orf1a* [16,18]. A failure of specific detection due to the sensitivity of the assay cannot be excluded. It is more likely that the all along low amount of MERS-CoV RNA was further decreased throughout the time period of storage and/or the transfer of the specimen. If the specimens would have originated from patients with confirmed exposure to MERS-CoV, these samples would be considered probable cases and reprocessing of the specimens would be highly recommended [19]. As the specimens that were retrospectively tested are from 2015 no additional specimen can be taken from the patients.

## Conclusion

The results from our study show that the RealStar MERS-CoV (N gene) RT-PCR kit 1.0 can be considered a suitable and reliable confirmatory assay in combination with the RealStar MERS-CoV RT-PCR kit 1.0 according to the diagnostic scheme recommended by WHO [6,17–19].

**Summary points**

- Emerging infectious diseases are becoming an increasingly important issue not only in tropic and subtropic regions but also in the northern hemisphere.
- Middle East respiratory syndrome coronavirus (MERS-CoV) was an unknown virus when it emerged in 2012, but immediate actions took place, including:
  - 2a: involvement of different specialized laboratories, sequencing of provided material, characterization of the virus and publication of the sequence data.
  - 2b: diagnostic methods could be developed based on these sequence data.
  - 2c: actions 2a and 2b lead to an immediate proposed diagnostics scheme by the WHO in order to assure accurate diagnostic of this novel virus.
- MERS-CoV is a rare disease, but a total number of 33 samples were tested at two different sites in Riyadh, Saudi Arabia; testing was done using all three proposed diagnostic reverse transcription-PCR targets.
- Storage and transport conditions have a major impact on sample quality and outcome of the diagnostics methods.
- The RealStar^®^ MERS-CoV (N gene) reverse transcription-PCR kit 1.0 can be considered a suitable and reliable confirmatory assay in combination with the RealStar MERS-CoV RT-PCR kit 1.0 according to the diagnostic scheme recommended by WHO.
